# The Continuing Burden of Advanced HIV Disease Over 10 Years of Increasing Antiretroviral Therapy Coverage in South Africa

**DOI:** 10.1093/cid/cix1140

**Published:** 2018-03-04

**Authors:** Meg Osler, Katherine Hilderbrand, Eric Goemaere, Nathan Ford, Mariette Smith, Graeme Meintjes, James Kruger, Nelesh P Govender, Andrew Boulle

**Affiliations:** 1 Centre for Infectious Diseases, Epidemiology and Research, School of Public Health and Family Medicine, University of Cape Town; 2 Médecins Sans Frontières, Southern African Medical Unit, Cape Town, South Africa; 3 HIV Department, World Health Organization, Geneva; 4 Department of Health, Provincial Government of the Western Cape; 5 Wellcome Centre for Infectious Diseases Research in Africa, Institute of Infectious Disease and Molecular Medicine, University of Cape Town; 6 Department of Medicine, University of Cape Town and Groote Schuur Hospital; 7 Centre for Healthcare-Associated Infections, Antimicrobial Resistance and Mycoses, National Institute for Communicable Diseases, National Health Laboratory Service; 8 Faculty of Health Sciences, University of the Witwatersrand, Johannesburg, South Africa

**Keywords:** antiretroviral therapy, CD4 count, morbidity, advanced HIV disease, South Africa

## Abstract

**Background:**

Antiretroviral treatment (ART) has been massively scaled up to decrease human immunodeficiency virus (HIV)–related morbidity, mortality, and HIV transmission. However, despite documented increases in ART coverage, morbidity and mortality have remained substantial. This study describes trends in the numbers and characteristics of patients with very advanced HIV disease in the Western Cape, South Africa.

**Methods:**

Annual cross-sectional snapshots of CD4 distributions were described over 10 years, derived from a province-wide cohort of all HIV patients receiving CD4 cell count testing in the public sector. Patients with a first CD4 count <50 cells/µL in each year were characterized with respect to prior CD4 and viral load testing, ART access, and retention in ART care.

**Results:**

Patients attending HIV care for the first time initially constituted the largest group of those with CD4 count <50 cells/µL, dropping proportionally over the decade from 60.9% to 26.7%. By contrast, the proportion who were ART experienced increased from 14.3% to 56.7%. In patients with CD4 counts <50 cells/µL in 2016, 51.8% were ART experienced, of whom 76% could be confirmed to be off ART or had recent viremia. More than half who were ART experienced with a CD4 count <50 cells/µL in 2016 were men, compared to approximately one-third of all patients on ART in the same year.

**Conclusions:**

Ongoing HIV-associated morbidity now results largely from treatment-experienced patients not being in continuous care or not being fully virologically suppressed. Innovative interventions to retain ART patients in effective care are an essential priority for the ongoing HIV response.

In the last decade, the massive scale-up of antiretroviral therapy (ART) services in sub-Saharan Africa has increased access to treatment, aiming to decrease human immunodeficiency virus (HIV)–related morbidity and mortality and, more recently, HIV transmission [1]. The initial focus of ART scale-up was to reduce severe morbidity and mortality; patients with an AIDS-defining condition or a CD4^+^ T-lymphocyte (CD4) count <200 cells/µL were therefore considered eligible for ART [2]. In response to successes in ART scale-up globally as well as increasing evidence of the benefits of earlier ART initiation, CD4 count eligibility thresholds have increased [3, 4], culminating in the current guidance to “Treat all,” enabling HIV-infected people to start treatment regardless of CD4 count [5, 6]. South Africa implemented this guidance on 1 September 2016 [7]. As the proportion of ART-eligible patients who are on ART increases, we would expect decreases in morbidity and mortality at a population level. In resource-limited settings, HIV-associated morbidity and mortality have nevertheless remained considerable [8].

Approximately 420000 people out of a total population of 6.3 million were projected to be living with HIV in the Western Cape province of South Africa in 2016. Approximately half of all HIV-infected individuals and >85% of those with a CD4 count <200 cells/µL were estimated to be on ART [9]. The current study was prompted by HIV clinicians reporting anecdotal concern that the number of hospitalized cases of cryptococcal meningitis, typically associated with late presentation and advanced immunodeficiency [10], was not declining over time as would be expected given increasing ART coverage (Figure 1) [11]. There is a concern that at a population level, the decline in the number of patients presenting for the first time with advanced immunodeficiency could be offset by an increasing number of patients on long-term ART who have interrupted, stopped, or failed on therapy [12].

**Figure 1.**
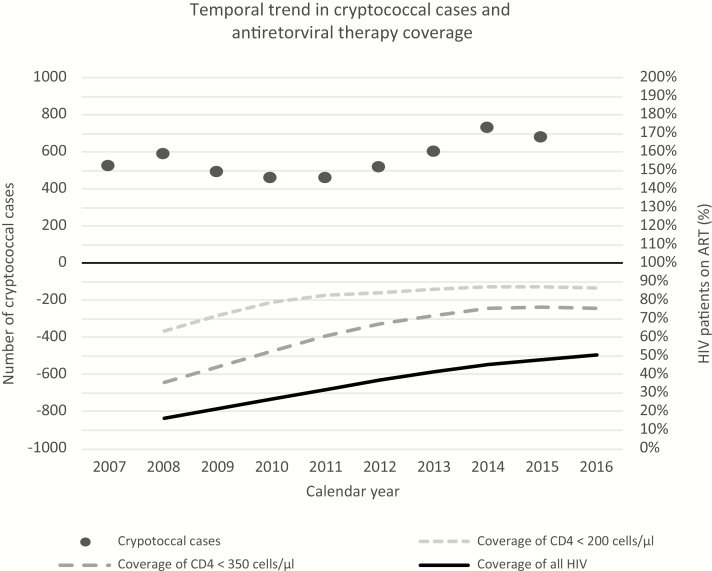
Temporal trends in the number of cases of laboratory-confirmed cryptococcosis and antiretroviral therapy (ART) coverage in patients with human immunodeficiency virus (HIV), Western Cape province, South Africa, 2007–2016.

This study describes temporal trends in the ascertainment of very advanced HIV disease in the Western Cape province of South Africa, evidenced by CD4 counts <50 cells/µL, and trends in the HIV treatment history of those with very advanced HIV disease.

## METHODS

### Setting and Data Sources

The Western Cape is one of 9 provinces in South Africa. One in 5 pregnant women has HIV. The vast majority of people living with HIV seek care in the public sector, which first offered ART in 2001, accelerating coverage after 2004 when ART provision became national policy [13]. CD4 monitoring of all patients and viral load monitoring once on ART have been provided since program inception but, since 2006, baseline viral loads were no longer recommended among ART-naive adult patients. From mid-2013, continued CD4 count monitoring among virologically suppressed and clinically well patients with CD4 ≥200 cells/µL at 1 year on ART was no longer recommended. The first-line regimen consists of 2 nucleoside reverse transcriptase inhibitors and a nonnucleoside reverse transcriptase inhibitor. Full details of the ART program evolution are described elsewhere [14].

All public sector laboratory testing is done by the National Health Laboratory Service (NHLS) and the digitized results have been available to the province since 2007. The province has successfully established a patient registration system which shares a unique health identifier and Patient Master Index (PMI) across both hospital and ambulatory services, which reached near-complete coverage as of 2013 [15]. This has facilitated the linkage of data from hospital, laboratory, and pharmacy sources, as well as electronic disease registers such as those for HIV and tuberculosis [16]. The process of linking all data to the PMI is now formalized through the Provincial Health Data Centre (PHDC), from which all data provided for analyses are preanonymized but still linkable based on a privacy-preserving random key. Cases of cryptococcosis were ascertained through routine laboratory-based surveillance for communicable diseases (GERMS-SA) conducted by the National Institute for Communicable Diseases based on NHLS data [17].

### Analysis

Annual cross-sectional snapshots of CD4 distributions were described from a derived province-wide HIV cohort of all patients receiving CD4 testing. Further longitudinal analyses focused on CD4 testing and ART treatment history in adults (≥16 years old) who were identified with CD4 <50 cells/µL in each period.

The cohort is based on data linkage between the laboratory-reported CD4 and viral load tests, and HIV treatment information systems. Data which had been previously linked for HIV operational reporting purposes were used for analyses until the end of 2012. Analyses for subsequent years were based on anonymized extracts from the PHDC. In this period, 98.7% of CD4 counts could be linked to the PMI.

In the manually linked database, the CD4 and viral load data had been linked to each other and to the HIV treatment database, using a combination of probabilistic and deterministic linkage based on name, surname, date of birth, sex, facility, subdistrict, district, and clinic folder number. Fine Grain Record Linkage (FRIL) version 2.1.5 (Emory University) and Stata version 13.0 (StataCorp) software were used for linkage and analysis. A random sample of linkages was manually validated. These data were anonymized prior to use for this analysis. Data from both sources combined were available from 2007 until 20 September 2017, and for 2017, all counts were upweighted to represent a full year’s worth of data to retain comparability with previous years.

The data are presented in annual cohorts based on the year the CD4 count was obtained. Results from 2007 are omitted as prior CD4 count testing could not be determined. Province-wide CD4 counts are described as absolute counts of tests performed as well as by counts of unique individual patients receiving testing in each year. Unique patients per year with CD4 <50 cells/µL were further stratified into the following categories in relation to ART status: late presenters (presenting for their first ever CD4 test with CD4 <50 cells/µL); previously not eligible for ART (inadequate pre-ART care); previously eligible for ART (failed linkage to or initiation of ART care); and those with a low CD4 count after ART initiation. ART initiation was determined by an ART start date recorded in the provincial electronic HIV registers or a viral load result, in which case the ART treatment start date was estimated as 4 months prior to the first recorded viral load (clinical guidelines prescribe the first viral load 4–6 months after treatment initiation). Patients were deemed eligible for ART if their CD4 was <200 cells/µL prior to 1 May 2010 or <350 cells/µL after 1 May 2010 as per operative national guidelines.

ART-experienced patients in whom visit-level treatment records were available were further stratified into 4 categories: lost to follow-up (LTFU; no antiretroviral medicines for ≥90 days) prior to their low CD4 count, including those who returned to care within 30 days prior to the low CD4 count but otherwise met this definition; those in care prior to the low CD4 count but who had had a recent (ending in the previous year) gap of >90 days without antiretroviral medicines; or continuously in care (no gaps >90 days without antiretroviral medicines in the year prior to the low CD4). Patients without medicine information linked to their visit were assumed to have been given 30 days of treatment and therefore were counted as lost to care at 120 days after their last appointment.

Within those in care (with or without gaps), patients were further stratified by those without viral load data, those with suppressed viral loads, those with viremia (1 viral load >1000 copies/mL), and those failing treatment (defined as 2 consecutive viral loads >1000 copies/mL prior to the low CD4 count).

Results of hypothesis testing for highlighted differences in proportions or tests for trend are not presented due to the large numbers of patients involved and universally low *P* values (all <.001). Age, duration on ART, and time between tests were described as medians and interquartile ranges.

The study was approved by the University of Cape Town Human Research Ethics Committee (421/2016).

## RESULTS

Over the 10-year period from 2008 through 2017, the total number of patients estimated to be living with HIV in the Western Cape increased to >400000, with the proportion estimated to be on ART increasing from 16% in 2008 to 52% in 2016 (Table 1). CD4 counts performed annually in adults rose over time, reaching close to 300000 tests per year in 2015, declining thereafter as routine annual CD4 monitoring was stopped. Clinical cryptococcosis cases remained >400 per year from 2005 to 2015 (Figure 1).

**Table 1.** Western Cape Human Immunodeficiency Virus Prevalence, Treatment Coverage and Counts of CD4 Assessments in Adults >16 Years

	2008		2009		2010		2011		2012		2013		2014		2015		2016		2017
WC population^a^	5 514 490		5 614 808		5 714 506		5 814 411		5 947 198		6 016 926		6 116 300		6 200 100		6 293 200		6 510 300
HIV prevalence in the Western Cape^b^, N (%)	278 083	(5.0%)	298 139	(5.3%)	317 132	(5.5%)	334 927	(5.8%)	353 577	(5.9%)	371 545	(6.2%)	388 786	(6.4%)	405 532	(6.5%)	421 751	(6.7%)	Not available
ART coverage based on CD4 <200; >14 years old^b^, %	63.4%		72.1%		78.9%		83.1%		84.4%		86.0%		87.5%		87.6%		86.9%		
ART coverage based on CD4 <350; >14 years old^b^, %	35.9%		44.3%		52.5%		60.8%		67.3%		72.1%		75.7%		76.2%		75.6%		
ART coverage (UTT); adults and children^b^, %	16.6%		21.5%		26.6%		32.1%		37.0%		41.5%		45.2%		48.3%		50.5%		
Total cases of cryptococcosis	591		489		460		460		519		598		729		677		Not available		Not available
Number of CD4 counts done (≥16 years)	172 618		215 425		226 435		258 381		258 547		251 030		269 833		296 282		207 058		183 384
Number of unique patients with CD4 counts done	117 758		142 397		161 396		185 601		191 587		201 280		213 616		213 388		177 356		166 822
Median age (N, [IQR])	33 [28–39]		34 [27–39]		34 [27–40]		34 [28–41]		34 [29–41]		39 [33–45]		38 [32–45]		37 [31–44]		36 [30–43]		35 [29–42]
Proportion male, %	30.2%		31.2%		31.3%		31.7%		31.9%		32.8%		33.3%		34.0%		35.3%		35.9%
Lowest CD4 value (cells/µL) in unique patients, N (%)																			
CD4 ≤ 50	7530 (6.4%)		8333 (5.9%)		7921 (4.9%)		7702 (4.1%)		7583 (4.0%)		7523 (3.7%)		8040 (3.8%)		9019 (4.2%)		8921 (5.0%)		8779 (5.3%)
CD4 51–100	6747 (5.7%)		7611 (5.3%)		7667 (4.8%)		6642 (3.6%)		6668 (3.5%)		7746 (3.8%)		8202 (3.8%)		8829 (4.1%)		8606 (4.9%)		8608 (5.2%)
CD4 101–200	24 872 (21.1%)		27 903 (19.6%)		29 185 (18.1%)		27 163 (14.6%)		24 869 (13.0%)		20 249 (10.1%)		20 824 (9.7%)		22 385 (10.5%)		20 620 (11.6%)		20 467 (12.3%)
CD4 201–350	35 687 (30.3%)		43 723 (30.7%)		50 004 (31.0%)		52 304 (28.2%)		51 522 (26.9%)		46 293 (23.0%)		46 148 (21.6%)		45 641 (21.4%)		37 913 (21.4%)		35 268 (21.1%)
CD4 351–500	22 447 (19.1%)		29 082 (20.4%)		34 755 (21.5%)		43 826 (23.6%)		47 295 (24.7%)		49 859 (24.8%)		51 669 (24.2%)		51 897 (24.3%)		39 091 (22.0%)		34 561 (20.7%)
CD4 > 500	20 475 (17.4%)		25 745 (18.1%)		31 864 (19.7%)		47 964 (25.8%)		53 650 (28.0%)		69 610 (34.6%)		78 733 (36.9%)		75 617 (35.4%)		62 205 (35.1%)		59 139 (35.5%)

Abbreviations: ART, antiretroviral therapy; IQR, inter quartile range; N, number; UTT, Universal Test and Treat.

^a^Census data and STATSSA mid-year population estimates.

^b^Thembisa model (2017 omitted as not calibrated).

**Figure 2.**
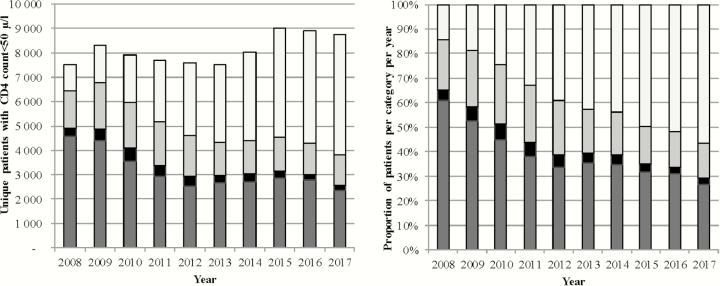
Presentation of unique patients with CD4 count <50 cells/µL by previous antiretroviral therapy access, eligibility, and previous CD4 cell count testing, stratified by year, Western Cape province, South Africa.

The number of unique patients per year with a lowest CD4 count test result <50 cells/µL varied between 6164 and 8133, remaining relatively constant over time (Table 1 and Figure 2). These patients were more likely to be men when compared to all patients receiving CD4 count testing, consistent over all years (Table 2).

**Table 2.** Characteristics of Adults With a CD4 Count Below 50 Cells/µL in the Western Cape

	2008		2009		2010		2011		2012		2013		2014		2015		2016		2017	
Unique patients with CD4 counts <50 , N (%)	7530		8333		7921		7702		7583		7523		8040		9019		8921		8779	
Median age (N, IQR)	34.3	[29–40]	34.4	[29–40]	34.7	[29–42]	35.1	[30–42]	35.3	[30–42]	3748	[34–45]	38	[33–45]	38	[32–44]	37	[32–43]	36	[31–43]
Men, %	45.6%		46.6%		46.7%		47.1%		49.3%		49.8%		51.0%		51.2%		51.1%		51.9%	
CD4 testing and ART history, N (%)																				
A. First ever presentation for CD4 count	4587	(60.9%)	4387	(52.6%)	3563	(45.0%)	2931	(38.1%)	2541	(33.5%)	2659	(35.3%)	2710	(35.7%)	2855	(31.7%)	2764	(31.0%)	2346	(26.7%)
B. Previously ART ineligible	326	(4.3%)	467	(5.6%)	524	(6.6%)	429	(5.6%)	388	(5.1%)	317	(4.2%)	316	(4.2%)	294	(3.3%)	240	(2.7%)	205	(2.3%)
C. Previously ART eligible, never on ART	1539	(20.4%)	1913	(23.0%)	1893	(23.9%)	1809	(23.5%)	1700	(22.4%)	1340	(17.8%)	1371	(18.1%)	1396	(15.5%)	1293	(14.5%)	1248	(14.2%)
D. Started ART prior to low CD4	1078	(14.3%)	1566	(18.8%)	1941	(24.5%)	2533	(32.9%)	2954	(39.0%)	3207	(42.6%)	3643	(48.0%)	4474	(49.6%)	4624	(51.8%)	4980	(56.7%)
Days since last CD4, median (IQR)																				
B. Previously ART ineligible	276	[144–394]	379	[182–657]	594	[325–899]	859	[443–1218]	1023	[520–1442]	1624	[1141–1973]	1699	[1199–2248]	2085	[1345–2514]	2416	[1767–2909]	2706	[2078–3126]
C.Previously ART eligible, never on ART	210	[75–348]	270	[102–512]	357	[138–694]	402	[173–854]	539	[247–914]	667	[281–1134]	740	[292–1285]	736	[264–1434]	878	[355–1676]	890	[340–1644]
Available ART visit-level records, N (%)	524	(48.6%)	850	(54.3%)	1116	(57.5%)	1495	(59.0%)	1883	(63.7%)	3206	(100.0%)	3643	(100.0%)	4474	(100.0%)	4623	(100.0%)	2231	(44.8%)
Retention in ART care prior to presentation in (D), N (%)																				
1 Considered LTF at last visit	118	(22.5%)	186	(21.9%)	297	(26.6%)	407	(27.2%)	593	(31.5%)	1275	(39.8%)	1514	(41.6%)	1936	(43.3%)	2109	(45.6%)	977	(43.8%)
2 In care but with gap (>180 days) in last year	34	(6.5%)	66	(7.8%)	106	(9.5%)	124	(8.3%)	137	(7.3%)	255	(8.0%)	339	(9.3%)	499	(11.2%)	477	(10.3%)	244	(10.9%)
3 Continuously in ART care during past year	372	(71.0%)	598	(70.4%)	713	(63.9%)	964	(64.5%)	1153	(61.2%)	1676	(52.3%)	1790	(49.1%)	2039	(45.6%)	2037	(44.1%)	1010	(45.3%)
Months since ART initiation in (D), median (IQR)																				
1 Considered LTF at last visit	21.8	[14.6–33.4]	27.9	[16.0–37.6]	29	[17.3–43.0]	32	[19.5–48.9]	32.6	[19.2–51.2]	36.5	[22.2–58.0]	42.2	[23.7–64.8]	44.8	[25.0–69.3]	49.6	[27.9–73.8]	51.7	[28.8–78.1]
2 In care but with gap (>180 days) in last year	28.1	[19.3–36.7]	26	[18.6–35.7]	31.3	[21.2–42.7]	34.8	[23.0–51.2]	38.6	[24.4–54.9]	39.2	[26.2–61.2]	43.4	[29.2–66.9]	49.4	[32.1–73.4]	51.2	[32.2–79.6]	57.4	[34.5–79.1]
3 Continuously in ART care during past year	12.1	[6.0–24.0]	14	[6.0–28.3]	17.7	[6.5–34.7]	17.2	[7.1–37.2]	20.3	[8.5–43.2]	25.4	[10.7–51.3]	33.3	[12.4–58.8]	37.2	[13.2–68.1]	43	[15.5–76.2]	46.8	[18.2–83.0]
Presentation in hospital in those LTF (1), %	47.5%		36.0%		31.0%		29.2%		33.1%		31.1%		28.8%		26.6%		29.5%		30.7%	
Virologic status in those with a gap in care (2), %																				
No data in the previous 15 mo	20	(58.8%)	40	(60.6%)	51	(48.1%)	71	(57.3%)	67	(48.9%)	91	(35.7%)	125	(36.9%)	203	(40.7%)	208	(43.6%)	84	(34.4%)
Supressed <1000 copies/mL	3	(8.8%)	9	(13.6%)	13	(12.3%)	5	(4.0%)	11	(8.0%)	21	(8.2%)	30	(8.8%)	41	(8.2%)	26	(5.5%)	18	(7.4%)
Viraemic just on previous test	6	(17.6%)	11	(16.7%)	28	(26.4%)	37	(29.8%)	38	(27.7%)	83	(32.5%)	76	(22.4%)	136	(27.3%)	114	(23.9%)	74	(30.3%)
Viraemic on previous two or more tests	5	(14.7%)	6	(9.1%)	14	(13.2%)	11	(8.9%)	21	(15.3%)	60	(23.5%)	108	(31.9%)	119	(23.8%)	129	(27.0%)	68	(27.9%)
Virologic status in those in continuous ART (3), %																				
No data in the previous 15 mo	210	(56.5%)	322	(53.8%)	336	(47.1%)	414	(42.9%)	462	(40.1%)	469	(28.0%)	499	(27.9%)	600	(29.4%)	571	(28.0%)	254	(25.1%)
Supressed <1000 copies/mL	68	(18.3%)	113	(18.9%)	120	(16.8%)	129	(13.4%)	150	(13.0%)	245	(14.6%)	336	(18.8%)	389	(19.1%)	286	(14.0%)	120	(11.9%)
Viraemic just on previous test	58	(15.6%)	93	(15.6%)	141	(19.8%)	243	(25.2%)	275	(23.9%)	448	(26.7%)	425	(23.7%)	476	(23.3%)	508	(24.9%)	281	(27.8%)
Viraemic on previous two or more tests	36	(9.7%)	70	(11.7%)	116	(16.3%)	178	(18.5%)	266	(23.1%)	514	(30.7%)	530	(29.6%)	574	(28.2%)	672	(33.0%)	355	(35.1%)

Abbreviations: ART, antiretroviral therapy; IQR, interquartile range; N, number.

Patients presenting for the first time initially constituted the largest group of those with very low CD4 counts, dropping proportionally over time from 60.9% to 26.7% (Figure 2 and Table 2). By contrast, the proportion of ART experienced patients increased from 14.3% to 56.7%. Throughout there was an appreciable proportion of patients who should have started ART as they had previously met ART eligibility criteria based on prior CD4 count testing; this declining from 20.4% to 14.2%. A much smaller proportion of patients were known to the services but were previously not ART eligible.

Of those ART naive with previous CD4 count testing prior to the index CD4 test (first CD4 <50 cells/µL per patient per year), the median time from the previous value to the index CD4 count increased over time. The time between consecutive CD4 counts was much longer in those who had previously been ART ineligible than those who had been eligible for ART (reaching more than 5 and 2 years in later years, respectively; Table 2).

By 2014, more than half of those who had previously been on ART prior to the index CD4 had either dropped out of care or had had a substantial gap in care in the previous year, reaching 45.6% and 10.3%, respectively, in 2016 (Table 2). The remainder had evidence suggesting they were on ART continuously for at least the year prior to the index CD4. For all groups previously on ART, the median duration between ART initiation and the index CD4 increased over time, approaching 4 to 5 years since ART initiation by 2017, and being consistently shorter in those continuously on ART. Of those who had been LTFU prior to the index CD4 test, a substantial proportion (close to a third in recent years) were detected at hospitals rather than ART clinics.

For patients on ART at the time of the index CD4 test, those with a gap in care in the preceding year were less likely to have a viral load result available from the preceding 15 months (56.4% vs 72.0% in 2016), and less likely to be virologically suppressed if tested (9.7% vs 19.5% of those tested in 2016). A higher number of those on ART with viremia (>1000 copies) had evidence of sustained viremia over ≥2 or more (56% in 2016).

**Figure 3.**
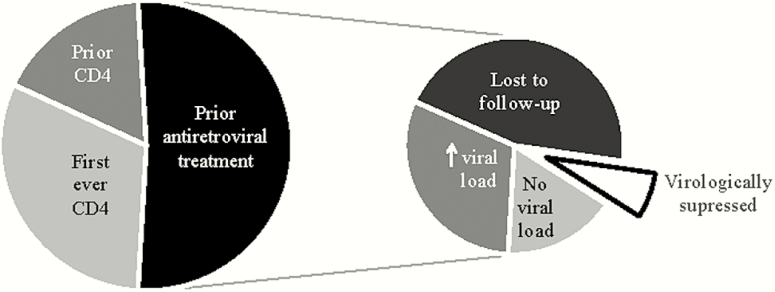
Distribution of prior CD4 count testing, antiretroviral therapy (ART), ART retention, and recent virologic status in patients presenting in 2016 with a CD4 count <50 cells/µL, Western Cape province, South Africa.

Looking across all patients with CD4 counts <50 cells/µL in 2016, 51.8% were ART experienced, among whom 76% could be confirmed to be off ART or had viremia at the time of the index CD4 test and 7% could be confirmed to be on suppressive ART; the virologic status of the remaining 17% was not known (Figure 3). The total number of patients with a CD4 count <50 cells/µL known to the services and lost to care or with viremia prior to the index CD4 test substantially exceeded the number not known to the services and presenting for the first time.

## DISCUSSION

This analysis has demonstrated how in a high-burden HIV setting, the proportion of patients presenting with very advanced HIV disease is not declining despite the successful scaling up of ART. This is reflected clinically by the relatively stable number of cases of cryptococcosis during the last decade, despite a considerable increase in access to ART. The proportion of patients initially presenting with very advanced HIV disease is declining, reflecting increased access to treatment; however, this reduction is being offset by an increasing number of patients who have previously started ART and are re-presenting with advanced HIV following a period without effective ART. Most often this is due to predictable reasons such as stopping ART, poor adherence, or virological failure, many of which are amenable to intervention.

### Ongoing HIV-Associated Morbidity

The initial interest from hospital-based clinicians in the province in the pattern of presentation with very low CD4 counts was in response to ongoing morbidity from conditions such as cryptococcal meningitis, which are associated with advanced immunodeficiency. Clinician-triggered and, more recently, laboratory-triggered reflex screening for cryptococcal antigenemia was introduced in 2014 and 2016, respectively, for all patients with CD4 counts <100 cells/µL. Although this likely prevents cryptococcal meningitis in some patients [18], there has been a disconcertingly high ongoing incidence since the alarm was first raised [11]. This is corroborated by surveys of HIV-associated morbidity in inpatient wards, where tuberculosis has been a major contributor to clinical presentation [19]. There is growing evidence that the successes of HIV treatment access are resulting in a sizeable population of individuals on ART who are vulnerable to rapid deterioration as they cycle in and out of care, as many do, owing to both service and personal challenges [12, 20].

### Where to Focus Interventions

The 90-90-90 strategy has successfully focused energy on identifying patients with HIV and linking them to care, bolstered by “Treat-all” guidelines. Many organizations have, as a result, concentrated in recent years on the identification of those with undiagnosed HIV and on improved linkage to care. The declining number of patients presenting for the first time in this study with advanced HIV disease points to the success of expanded access to HIV testing, care, and treatment. In many settings, the 90% target for diagnosing HIV in those infected is close to achievement [21]. The dominant contribution of ART-experienced patients to advanced disease in this study cautions against complacency and suggests similar efforts be directed to ensuring adherence and retention within ART programs. Modeling suggests that, even for transmission, the variable most associated with future transmission is ensuring virologic suppression in those already on ART [22].

### Identifying in Which Patients to Intervene

Our findings point to extensive missed opportunities for intervention in patients who had started ART. The majority had either fallen out of care where reengagement interventions could have been triggered by missed appointments or formally defined LTFU, or had previously had viremia or had missed viral load testing. Of those with viremia, half had been confirmed to be so on >1 consecutive occasion. Clinical governance and quality-improvement processes could address the efficient implementation of guidelines for virologic testing, and adherence promotion and regimen switching in patients fulfilling virological failure criteria. The increasing affordability and tolerability of new regimens should remove concerns around premature switching, and the balance should shift to more aggressive intervention to ensure suppressive ART. The study also corroborated previous findings of excess mortality risks in men [23] which, although not limited to HIV outcomes, might guide the design of some interventions.

### Surveillance of Advanced Disease

“Community viral load” tracks transmission potential [24, 25], but does not describe the immunological status of patients. Routine CD4 monitoring is no longer recommended in virologically suppressed patients [26], undermining the validity of CD4 count distribution from routine data as a population-level barometer of treatment success. The absolute numbers of patients with very low CD4 counts might, however, remain a sensitive barometer, where patients will often still be tested for advanced immunodeficiency for clinical reasons. Specific HIV-associated morbidities, hospital admissions, or mortality could also be used as measures of advanced disease. The advantage of a laboratory marker is that it may be easier to secondarily derive at scale across whole jurisdictions, as digitizing and centralizing of laboratory data are in any case operationally required. We propose that “community CD4 count,” represented in a dichotomized way by the absolute numbers and proportions of patients with very low CD4 counts, may be an accessible and reliable indicator of advanced HIV disease. This is akin to, in the case of viral load, assessing the proportion of patients who are confirmed or presumed to be viremic, as a dichotomized proxy for transmission potential.

### Limitations

There are several limitations to this study. Two separate data linkage exercises were stitched together to create a 10-year trend history, and while they aligned remarkably well at the seam, it would have been preferable to have a single approach. There may have been residual failures to link observations due to incomplete data for patient matching, either missing antecedent CD4 count histories or subsequent ART initiation. In terms of the major findings from this study, however, both biases, if present, would result in underestimation rather than overestimation of the trends toward fewer presentations in newly diagnosed patients and more in care- or treatment-experienced patients. Some of the trends may further be artefact of the availability of data only from 2007 onward, especially durations, as previous measures, by design, should get longer with increasing calendar time due to the lack of prior data in the early years of the analysis.

## CONCLUSIONS

This study has demonstrated that ongoing HIV-associated morbidity now results largely from treatment-experienced patients not being in continuous care or not maintaining virologic suppression. Attention of healthcare services will need in the future to focus much more aggressively on the innovation and investment in and quality of ART services in order to avert transmission, morbidity, and mortality. In this study we have shown that monitoring the absolute numbers of very low CD4 counts at a population level over 10 years provided a readily accessible, durable, and sensitive integrated indicator of overall program performance and identified specific program deficiencies.

## Notes

***Acknowledgments.*** The authors thank Leigh Johnson for calculating and sharing the estimates of ART coverage.

***Disclaimer.*** The funders had no role in the study design, data collection, data analysis, data interpretation, or writing of this report. The opinions, findings and conclusions expressed in this manuscript reflect those of the authors alone.

***Financial support.*** G. M. was supported by the Wellcome Trust (grant number 098316); the South African Research Chairs Initiative of the Department of Science and Technology and National Research Foundation (NRF) of South Africa (grant number 64787); NRF incentive funding (unique identifier [UID]: 85858); and the South African Medical Research Council through its TB and HIV Collaborating Centres Programme with funds received from the National Department of Health (RFA number SAMRC-RFA-CC: TB/HIV/AIDS-01-2014). A. B. was supported by the National Institutes of Health (grant numbers R01 HD080465, U01 AI069924, R01 MH106600); the Bill & Melinda Gates Foundation (grant number OPP1164272 and HIV Measurement and Surveillance Consortium [MeSH]); and NRF incentive funding (UID: 103528). This supplement was supported by funds from the Bill & Melinda Gates Foundation.

***Supplement sponsorship.*** This article appears as part of the supplement “Advanced HIV Disease,” sponsored by the World Health Organization.

***Potential conflicts of interest.*** All authors: No reported conflicts of interest. All authors have submitted the ICMJE Form for Disclosure of Potential Conflicts of Interest. Conflicts that the editors consider relevant to the content of the manuscript have been disclosed.

## References

[1] World Health Organization. Progress report 2016, Prevent HIV, Test and Treat All, WHO support for country impact. Geneva, Switzerland: WHO, 2016.

[2] World Health Organization/Joint United Nations Programme on HIV/AIDS. Treating 3 million people living with HIV/AIDS by 2005: Making it Happen: the WHO strategy. The WHO and UNAIDS global initiative to provide antiretroviral therapy to 3 million people with HIV/AIDS in developing countries by the end of 2005. Geneva, Switzerland: WHO, 2004.

[3] World Health Organization. Rapid advice: antiretroviral therapy for HIV infection in adults and adolescents. Geneva, Switzerland: WHO, 2009.

[4] World Health Organization. Consolidated guidelines on the use of antiretroviral drugs for treating and preventing HIV infection: recommendations for a public health approach. Geneva, Switzerland: WHO, 2013:272. 24716260

[5] World Health Organization. Guideline on when to start antiretroviral therapy and on pre-exposure prophylaxis for HIV. Geneva, Switzerland: WHO, 2015. 26598776

[6] World Health Organization. Guidelines for managing advanced HIV disease and rapid initiation of antiretroviral therapy. Geneva, Switzerland: WHO, 2017. 29341560

[7] South Africa National Department of Health. Implementation of the universal teat and treat strategy for HIV positive patients and differentiated care for stable patients. Pretoria: South Africa National Department of Health, 2016.

[8] Gabillard D, Lewden C, Ndoye I et al ANRS 12222 Morbidity Mortality Study Group Mortality, AIDS-morbidity, and loss to follow-up by current CD4 cell count among HIV-1-infected adults receiving antiretroviral therapy in Africa and Asia: data from the ANRS 12222 collaboration. J Acquir Immune Defic Syndr 2013; 62:555–61. 23274931 10.1097/QAI.0b013e3182821821 PMC3921662

[9] University of Cape Town Thembisa model, version 2.5. 2017 Available at: https://www.thembisa.org. Accessed 21 September 2017.

[10] Montlahuc C, Guiguet M, Abgrall S et al French Hospital Database ANRS CO4 cohort Impact of late presentation on the risk of death among HIV-infected people in France (2003-2009). J Acquir Immune Defic Syndr 2013; 64:197–203. 24047970 10.1097/QAI.0b013e31829cfbfa

[11] Jarvis J, Meintjes G Cryptococcal meningitis—a neglected killer. S Afr Med J 2011; 101:244–5. 21786726 10.7196/samj.4795

[12] Kaplan S, Oosthuizen C, Stinson K et al Contemporary disengagement from antiretroviral therapy in Khayelitsha, South Africa: a cohort study. PLoS Med 2017; 14:e1002407. 29112692 10.1371/journal.pmed.1002407 PMC5675399

[13] Bekker LG, Venter F, Cohen K et al Provision of antiretroviral therapy in South Africa: the nuts and bolts. Antivir Ther 2014; 19(Suppl 3):105–16. 25310359 10.3851/IMP2905

[14] Stinson K, Goemaere E, Coetzee D et al Cohort profile: the Khayelitsha antiretroviral programme, Cape Town, South Africa. Int J Epidemiol 2017; 46:e21. 27208042 10.1093/ije/dyw057

[15] World Health Organization. Consolidated guidelines on person-centred HIV patient monitoring and case surveillance. Geneva, Switzerland: WHO, 2017.

[16] Osler M, Hilderbrand K, Hennessey C et al A three-tier framework for monitoring antiretroviral therapy in high HIV burden settings. J Int AIDS Soc 2014; 17:18908. 24780511 10.7448/IAS.17.1.18908 PMC4005043

[17] National Institute of Communicable Diseases. GERMS-SA annual report 2015. Johannesburg, South Africa: NICD, 2015.

[18] Vallabhaneni S, Longley N, Smith M et al Implementation and operational research: evaluation of a public-sector, provider-initiated cryptococcal antigen screening and treatment program, Western Cape, South Africa. J Acquir Immune Defic Syndr 2016; 72:e37–42. 26926942 10.1097/QAI.0000000000000976 PMC4871265

[19] Meintjes G, Kerkhoff AD, Burton R et al HIV-related medical admissions to a South African district hospital remain frequent despite effective antiretroviral therapy scale-up. Medicine (Baltimore) 2015; 94:e2269. 26683950 10.1097/MD.0000000000002269 PMC5058922

[20] Stern E, Colvin C, Gxabagxaba N, Schutz C, Burton R, Meintjes G Conceptions of agency and constraint for HIV-positive patients and healthcare workers to support long-term engagement with antiretroviral therapy care in Khayelitsha, South Africa. Afr J AIDS Res 2017; 16:19–29. 28367748 10.2989/16085906.2017.1285795 PMC5557274

[21] Johnson LF, Rehle TM, Jooste S, Bekker LG Rates of HIV testing and diagnosis in South Africa: successes and challenges. AIDS 2015; 29:1401–9. 26091299 10.1097/QAD.0000000000000721

[22] Johnson LF, Chiu C, Myer L et al Prospects for HIV control in South Africa: a model-based analysis. Glob Health Action 2016; 9:30314. 27282146 10.3402/gha.v9.30314 PMC4901512

[23] Cornell M, Schomaker M, Garone DB et al International Epidemiologic Databases to Evaluate AIDS Southern Africa Collaboration Gender differences in survival among adult patients starting antiretroviral therapy in South Africa: a multicentre cohort study. PLoS Med 2012; 9:e1001304. 22973181 10.1371/journal.pmed.1001304 PMC3433409

[24] Kranzer K, Lawn SD, Johnson LF, Bekker LG, Wood R Community viral load and CD4 count distribution among people living with HIV in a South African township: implications for treatment as prevention. J Acquir Immune Defic Syndr 2013; 63:498–505. 23572010 10.1097/QAI.0b013e318293ae48 PMC4233323

[25] Herbeck J, Tanser F Community viral load as an index of HIV transmission potential. Lancet HIV 2016; 3:e152–4. 27036988 10.1016/S2352-3018(16)00036-9

[26] Ford N, Meintjes G, Pozniak A et al The future role of CD4 cell count for monitoring antiretroviral therapy. Lancet Infect Dis 2015; 15:241–7. 25467647 10.1016/S1473-3099(14)70896-5

